# CHARACTERIZATION OF THE PSYCHOLOGICAL TYPOLOGY IN ESOPHAGEAL CANCER PATIENTS

**DOI:** 10.1590/0102-672020220002e1715

**Published:** 2023-01-09

**Authors:** Stela Duarte PINTO, Lórgio Henrique Diaz RODRIGUEZ, Flávio Roberto TAKEDA, Marcos Roberto TACCONI, Rubens Antonio Aissar SALLUM, Ivan CECCONELLO, Ulysses RIBEIRO

**Affiliations:** 1 Universidade de São Paulo, Faculty of Medicine, Cancer Institute of São Paulo State, University Hospital, Psichology Unit – São Paulo (SP), Brazil;; 2 Universidade de São Paulo, Faculty of Medicine, Cancer Institute of São Paulo State, University Hospital, Gastroenterology Department – São Paulo (SP), Brazil.

**Keywords:** Personality, Esophageal Neoplasms, Psychological Tests, Psychological Theory, Surgical Oncology, Personalidade, Neoplasias Esofágicas, Testes Psicológicos, Oncologia Cirúrgica

## Abstract

**BACKGROUND:**

Esophageal cancer is an environment-related disease, and the most important risk factors are alcohol intake and smoking, in addition to gastroesophageal reflux in obese patients. The characterization of the patients’ personality can contribute to the perception of how everyone adapts to the social environment and what relationship one can establish with themselves and with others.

**AIM:**

The aim of this study was to identify the psychological typology in patients with esophageal cancer.

**METHODS:**

The psychological typology of patients was defined using the Typological Assessment Questionnaire. In addition, the aspects of psychological assessment were studied to access the particularities of each patient, especially their reaction to the diagnosis and the meaning attributed to the disease.

**RESULTS:**

A total of 90 patients with esophageal cancer, aged over 18 years, who completed high school, and were interviewed at the first medical appointment, were included. The introverted attitude was predominant (83.33%). The most common psychological type was introverted sensation, with feeling as a secondary function (43.3%), and the second most frequent was introverted feeling, with sensation as a secondary function (24.4%). From this psychological assessment, a variety of defensive mechanisms were found to minimize distress. Most patients made use of adaptive defenses in the face of the illness process.

**CONCLUSION:**

The identification of the psychological typology allows the most effective assistance in directing the peculiar needs of each patient. In addition, it contributes to the care team to individualize treatments based on specific psychological characteristics.

## RESUMO

**RACIONAL:**

O câncer de esôfago é uma doença relacionada ao meio ambiente, e os fatores de risco mais importantes são a ingestão de álcool e tabagismo, além do refluxo gastroesofágico em pacientes obesos. A caracterização da personalidade do paciente contribui para a percepção de como cada indivíduo se adapta ao meio social e que relação pode estabelecer consigo mesmo e com os outros.

**OBJETIVO:**

Identificar a tipologia psicológica em pacientes com câncer de esôfago.

**MÉTODOS:**

Definiu-se a tipologia psicológica utilizando o Questionário de Avaliação Tipológica. Estudaram-se os aspectos da avaliação psicológica para acessar as particularidades de cada um dos pacientes, principalmente no que diz respeito à sua reação ao diagnóstico e ao significado atribuído à doença.

**RESULTADOS:**

Foram incluídos noventa pacientes, com mais de 18 anos, ensino médio completo, diagnóstico de câncer de esôfago e que foram entrevistados na primeira consulta médica. A atitude introvertida foi a preponderante (83,33%). O tipo psicológico mais comum foi introvertido — sensação, tendo o sentimento como função secundária (43,3%), e introvertido — sentimento com sensação como função secundária foi o segundo tipo mais frequente (24,4%). A partir dessa avaliação psicológica, encontrou-se uma variedade de mecanismos defensivos para minimizar a angústia. A maioria dos pacientes fez uso de defesas adaptativas diante do processo de adoecimento.

**CONCLUSÕES:**

A identificação da tipologia psicológica permite o auxílio mais eficaz no direcionamento das necessidades peculiares de cada indivíduo. Além disso, contribui com a equipe de atendimento a fim de individualizar os tratamentos com base nas características psicológicas específicas.

## INTRODUCTION

Despite considerable technological, surgical, and pharmacological advances, cancer is still considered a stigmatizing disease, which brings up the possibility of imminent death. The general population considers it a devastating disease, which (consciously) everyone wants to get away from. However, psychological theorists characterize diseases as a symbolic and compensatory expression of a one-sided conscious attitude^16^.

Esophageal cancer is a disease related to the environment, and the most important risk factors include alcohol ingestion and tobacco smoking, besides gastroesophageal reflux in obese patients^9,13^.

The characterization of the patients’ personalities can contribute to the perception of how everyone adapts to the social environment and what relationship one may establish with themselves and with others. Thus, getting a glimpse of the aspects of the inner world of these individuals can help us better understand how they perceive and interact with the outside world and may help them with therapeutic planning and management^2^.

Through detailed studies, Jung was able to show us concepts that facilitate the perception of the psychic structure of a person, referred to as psychological types^2,4^.

We can consider that psychological typology is the general layout of the human being, which remains identical under various conditions. Personality characteristics are not changeable according to the events of the individual’s life, since they are inherent to one’s personal development and path^6^. Typology does not aim at standardizing people. It is a critical investigative tool that offers a methodical sorting of psychic contents and helps in understanding of the individual diferences^4,14^.

Jung has described opposing attitudes according to the attention focus: extraversion and introversion^2^. In an extraverted person, a relationship is established between the subject and the object; the extraverted person adopts the point of view of most people. On the contrary, introversion is initially an internal movement, so the person with this attitude feels, thinks, and acts from their motivation^2,4,14^.

Furthermore, Jung described four psychological functions: thinking, feeling, intuition, and sensation^2-4^.

The objective of this study was to identify psychological typology in patients with esophageal cancer. Moreover, the emotional reactions of patients when facing the oncological diagnosis and what meaning they give to cancer in their lives were also examined. Finally, we propose, from this knowledge, an innovative and distinct treatment strategy for these patients.

## METHODS

This research was approved by the Ethics Committee of the University Hospital, Faculty of Medicine of University of São Paulo, under research protocol (no. 037/14). The patients signed the informed consent before the application of the instruments for data collection.

This was an exploratory, descriptive, and transversal study performed in a tertiary cancer hospital.

- **Inclusion criteria:** Patients over 18 years of age, with at least a middle-school level education (informed at the initial interview), who had been diagnosed with esophageal cancer and were met during their first medical appointment were included.
- **Exclusion criteria:** Patients with a history of diseases who would interfere with their understanding and ability to answer the instruments, as well as illiterate patients were excluded.
- **Study location:** Digestive Surgery Outpatient Clinic at the Cancer Institute of São Paulo State — University Hospital of the Faculty of Medicine, University of São Paulo (ICESP-HCFMUSP).
  The psychologist (SDP) presented the research and the study objectives to the patients, and if the patients agreed to participate in the survey, they were given the terms for signing the Free and Informed Consent, and the instruments for data collection were applied.
  Patients were evaluated in individual rooms, according to the routine of the Psychology Service Institution. During the first session, we aimed to know the patient’s psychological particularities, and they answered the Typological Assessment Questionnaire (QUATI).
- **Instrument description**: QUATI: a psychological test developed by Zacharias^15^.
  This test seeks to identify the person’s prevailing psychological type, that is, the attitude adopted by the subject and its relationship to the world and with other people, as well as the main and secondary psychological functions (Table 1).
  QUATI allows us to identify if the person is introverted or extroverted, if they prioritize a judgment or perception to interact with themselves, with people and events, as well as which one complements and supports the main function.
  This instrument is restricted to psychologists (as with all psychological tests). It cannot be included in this article entirely, as it is protected by copyright laws; one can only use it in its original version.

**Table 1.** Summary of the parameters evaluated by Typological Assessment Questionnaire^15^.

QUATI	Typological Evaluation Questionnaire	Author: Zacharias JJM^15^
93 questions	Contents of everyday situations	Correction examples below
**“A party”**(With a lot of people, some you know, others you do not know; there is a happy animated atmosphere in the room, music, and so you).	Pay attention to everything and everyone around you (extroversion); you are more inclined toward your own discomfort (introversion).Does not pay attention to the details of the party (intuition); especially aware of all the details and does not forget them (sensing).	How to define the results: below are the necessary guidelines.To correct this psychology test, you must use the specific correction grid, divided into three parts: R1–Ex × I; R2–In × Ss; R3–Ps × St.These three aspects are divided into two respective options, A and B; you should note the result, the sum of answers A and B.Afterward, subtract the lowest value from the highest value. The letter that obtains the highest points will be defined as R1, R2, and R3.Qualitative results are extracted and defined by code-letters found in R1, R2, and R3; and quantitative, considering the values found for R1, R2, and R3.Stemming from this, we already have a defined attitude of the subject (Ex or I).The main function will be the highest points between R2 and R3.The secondary function will be the one that receives the lowest number of points between R2 and R3.The lesser function will be the opposite one of the main function.
**“The job”**(Where your hierarchy position is a middle one, with superiors as well as employees under your supervision and orientation).	Pays close attention to know about everything that is going on around (extroversion; concentrates on work (introversion).Enjoys taking part in new activities and innovating projects (intuition).Likes routine and attracted by it (sensing).	
**“The trip”**(If it is a vacation where you will spend a month away from home).	Are more interested in sports activities (extroversion); are more interested in quiet walks (introversion).Live the expectancy of the trip more intensely (intuition); live the moment of the trip more intensely (sensing).	
**“Studying”**(In a classroom, with different subjects, among classmates).	Does better when presenting at a seminar (extroversion); does better writing an essay (introversion).Enjoy creative activities (intuition).Enjoy practical activities (sensing).	
**“Free time”**(Facing a weekend without any planned functions).	Prefers going to a talk (thinking).Prefers taking part in good conversations (feeling).Would refuse an invitation from a friend, explaining the reasons why (thinking); would find it difficult to refuse a friend’s invitation (feeling).	
**“Personal life”**(You consider yourself to be more).	Objective (thinking); kind (feeling).Impersonal and logic (thinking); personal and sentimental (feeling).	

QUATI: Typological Assessment Questionnaire.

### Statistical analysis

Sample size was calculated based on the definition of the number of treated esophageal cancer patients, annually, with 20% variation, 95% confidence interval level, and 5% margin of error or variance. Data analysis consisted of the construction of confidence intervals of 95% for the psychological type percentages, and a Fisher’s exact test was used to verify the association between variables of interest.

## RESULTS

Demographics show a higher prevalence of this tumor in males, in patients with only primary education, married, most having no cancer history, as well as any neoplasia family history. A high number of patients had a history of alcoholism and smoking or maintained such habits at the time of the data collection. Squamous cell carcinoma was the most frequent histological esophageal tumor type. The demographics data, associated with the focus of attention, introverted or extroverted attitude, are presented in Table 2.

**Table 2.** Distribution of patients according to typological attitude (focus of attention) and the relationship to the clinical variables.

		n=75 (83.33%)	n=15 (16.67%)	p-value
		INTROVERT	EXTROVERT	
Sex	Male	62 (82.67%)	14 (93.33%)	0.44
	Female	13 (17.33%)	1 (6.67%)	
Age	Mean	61.35	60.13	0.53
Schooling	Middle school	64 (85.33%)	7 (46.67%)	**0.007**
	High school incomplete	3 (4%)	3 (20%)	
	High school complete	5 (6.67%)	2 (13.33%)	
	Higher education incomplete	1 (1.33%)	2 (13.33%)	
	Complete higher education and graduate studies	2 (2.67%)	1 (6.67%)	
Marital status	Single	7 (9.33%)	3 (20%)	0.34
	Married	48 (64%)	11 (73.33%)	
	Divorced	15 (20%)	1 (6.67%)	
	Widowed	5 (6.67%)	0	
Personal	Yes	5 (6.67%)	1 (6.67%)	
History	No	70 (93.33%)	14 (93.33%)	
Family	Yes	28 (37.33%)	4 (26.67%)	0.55
History	No	47 (62.67%)	11 (73.33%)	
Alcoholism	Yes	1 (1.33%)	1 (6.67%)	0.42
	Ex-alcoholic	57 (76%)	11 (73.33%)	
	No	17 (22.67%)	3 (20%)	
Smoking	Yes	17 (22.67%)	3 (20%)	0.86
	Ex-smoker	43 (57.33%)	8 (53.33%)	
	No	15 (20%)	4 (26.67%)	
Histological	Squamous cell carcinoma	60 (80%)	10 (66.67%)	0.3
Type	Adenocarcinoma	15 (20%)	5 (33.33%)	

Bold indicates significant value.

The majority of these patients had an *introverted* attitude, 83.33%. Of the 16 possible psychological types, 14 were found in this study. The most frequent psychological type encompasses *sensation* as a main conscious function, present in 43.33% of the patients, and as a secondary function *feeling* observed in 64.1%, and *thinking* in 35.9% (Table 3).

**Table 3.** Distribution of the 90 patients according to the data obtained from the Typological Assessment Questionnaire^15^, regarding the conscious functions of the patients (main function and auxiliary function).

Functions of consciousness	Two of each patient
Sensation	72 (40%)
Intuition	18 (10%)
Thinking	31 (17.22%)
Feeling	59 (32.78%)

The second most frequent psychological type was a variation of the first, that is, *introverted feeling* with *sensation* as a secondary function.

Qualitative data were analyzed individually. In each report given by the patient, we sought to make a discourse analysis associated with the functions. In other words, from the patient’s answers, we dwelled on the implicit aspects of the mental functions and characteristics of the psychological types, later to be associated with the type defined through the QUATI assessment^15^. From there, we defined the synthesis of the psychological defenses adopted by each one facing the crisis generated by the disease, so that we understood the emotional path of the patient facing the illness process.

Some of the patients adopted characteristics of their dominant function and/or their secondary function to talk about their reaction and the understanding given to the disease; sometimes, such a stance revealed itself as being adaptive, with the use of appropriate strategies to deal with the anguish and envision ways of mitigating it. However, some patients, making use of such conscious functions, were evasive and came into less contact with the possible implications brought on by this experience.

In general, the vast majority of people adopted an active and assertive posture when facing the illness process, looking for internal resources, inherent to each one, in addition to an external aid for minimizing the suffering as well as ways to deal with the reality of life marked by the oncological disease.

Most patients (67.78%) expressed, through their speech, adapted defensive mechanisms to minimize the suffering unleashed by the disease, and for this, they used rationalization mechanisms. Another group of patients defended themselves through their conscious functions, main and/or secondary, while a large number succeeded in integrating the inferior, least developed, conscious function so that it contributed to helping them face the situation and decreasing anguish. The remainder integrated the tertiary function with consciousness to assist in addressing the situation.

The remainder of the patients, in a diversified manner, voiced fragile defensive mechanisms so as not to reduce the suffering experienced. Others made use of the defensive mechanisms to avoid the reality they experienced. They did this through repression of the anxiety-generating content in the unconscious; denial of the situation as presented to the subject; displacement by replacing behavior and thoughts with other more socially accepted ones; and projection by placing features, thoughts, and actions that we carry on others, but we do not want to accept as our own. In addition, because the lower function is integrated into the conscious, it is done in a nonadaptive way. These data are distributed in Table 4.

**Table 4.** Distribution of the 10 defense types identified in the esophageal cancer patients’ speech.

Lower adaptive function	18 (20%)
Adaptive defenses through conscience functions	18 (20%)
Defensive mechanism of rationalization	18 (20%)
Fragile defensive mechanisms	15 (16.67%)
Defensive mechanism of repression	8 (8.89%)
Tertiary function in adaptive mode	7 (7.78%)
Defensive mechanism of denial	3 (3.33%)
Defensive mechanism of displacement	1 (1.11%)
Defensive mechanism of projection	1 (1.11%)
Lower nonadaptive function	1 (1.11%)

## DISCUSSION

Over the course of human development and the start of psyche development, a person will acquire certain skills that lead to connecting with themselves and with the surrounding world, prioritizing a better adaptation to the environment. In this way, one of the attitudes will differ, as well as one of the functions, which will be considered the main or dominant function. This function is defined by a certain predisposition of the person and a genuine tendency to make use of their skills and aptitudes in such a way that it reveals the way they prefer to interact with everything surrounding them. Another function (auxiliary), called a secondary function, will be developed, but with less intensity^4,14,16^.

The secondary function enables a complement to the considerations of the main function; although it has a secondary role, its nature is different from the predominant one. In other words, every person has two secondary factors: a perception function and another one for judgment, which contributes to another point of view. There is no ambiguity between these functions and the direction of the subject is not confused, seeing that such functions are opposite in nature, with different, but complementary, roles in the psychic dynamics of each one. The main function will always be prioritized and will be the guiding function for the person^4,14^.

The two other functions will have less developed aspects and will remain unconscious. Because of this, one of them is called a tertiary function (of the same kind of secondary function, but contrary to it), and diametrically opposed to the main one, it is considered an inferior function^4,14^.

For individual attention, we have opposing attitudes: extraversion and introversion^5^. In extraversion, a relationship is established between the subject and the object; the person feels, thinks, and acts in relation to the object; it is a type of transfer from the subject to the object. Therefore, the extravert adopts the point of view of most people, because the focus is given to the object, the objective circumstances, and not from subjective opinions. On the contrary, the introversion is initially an internal movement, so that the person with this attitude feels, thinks, and acts from their motivation. In other words, the object has a secondary value since the focus is given to the subject. In this way, the introvert, being introspective, is more prone to auto-reflection and behaves abstractively^2,4,14^.

In addition to the attitudes that guide the person to relate with the environment, there are functions specific to each one, which determine the way the individual will manifest themselves. There are four psychological functions: thinking, feeling, intuition, and sensation^8^.

Thinking and feeling functions are rational and organizers of the subject. The thinking type establishes a connection between the concepts; it uses intellectual faculties to adapt to circumstances and live with people. On the contrary, the feeling type judges the value, having the capacity for weighing and evaluating an issue without analyzing their reason for it; it is a subjective process stemming from the person’s own referential^2,4,14^.

Intuition and sensation functions are identified as an involuntary, perceptive, and irrational act. Intuition is an instinctive adaptation that allows a person to realize their unconscious contents. The sensation type reveals perceived physical stimulus, causing stimuli in the body^3,4,16^.

The main function detected in this group of patients was sensation, and the secondary function was feeling. This shows that the study population based itself on the here-and-now, having trouble delaying gratification, emphasizing obtaining pleasure through the sense organs, and judging the world around them and their own tendencies by their judgment of values, for example, by what they judge as correct or not^14^.

The sensation function is considered the most common (75%) in the general population, which is used as a reference an American population study. In Brazil, the feeling function is the most frequent among the population^7^.

In this study, associating the main and secondary functions, we found *sensation* and *feeling* to be conscious predominant functions, data that converge with national and international realities.

The patients with the predominant psychological type in this study, i.e., introverted/sensation/feeling, indicate that they need explanations geared to a practical application without many abstractions, because it is easier for them to understand the sense of things stemming from what they usually do. These patients are auto-reflective and introspective, purely concrete. They live in the present intensely as if there were no prospects for future changes, enjoying the pleasures of the sense organs and having difficulty in postponing rewards^14^.

For this group of patients, it is important that the secondary function, feeling, continues to develop. This will allow them greater consistency in ideas and organization, as well as greater attention to the relevancy of the external aspects of the world around them^14^.

Patients with introverted/feeling/sensation, who make up the second largest group in this sample, are also reserved; they primarily evaluate the world around them based on their personal values and ideals, but they can be influenced by people who have a significant link to them; they exhibit tolerance and flexibility as long as their ideas are not threatened. They show difficulty in operationalizing what they had previously envisioned, so it becomes important to find a practical way to express their ideals^14^.

It is noteworthy to mention the importance of acceptance and certain bondage incorporated between the attending team and this group of patients, as they need to feel effectively involved with the team so they feel safe, because feeling-type people need to establish personal ties with their caregivers^11,14^.

By applying QUATI, we could identify the patient’s psychic structure, however with the illness they had already modified internally, because there was the willingness to access dynamic intra-psychic contents, favoring an integration of unconscious aspects with customary attitude resources and conscious functions. When strengthening healthy elements in our personality, we favor mobilizing unconscious aspects as possible resources for facing a crisis.

This fact brings us to resilience, which can be regarded as a person’s ability to deal with potentially distressing adverse situations in an adaptive way^1^.

Relating to psychosocial processes, they contribute to the sound development of the subject, favoring greater internal strengthening after experiencing a crisis. Due to the environment, the life cycle, and the vicissitudes of people, it is a dynamic and evolving process. When facing life’s events, greater flexibility and equilibrium become possible through resilience^1,10-13^.

Through the results found in this work, most patients are considered resilient, because a large part sought to learn from the disease and reflect on aspects of their lives, including adopted behaviors, at times even glimpsing certain transformations, such as alcoholism and smoking habits cessation. Another corroborating aspect relates to the fact that some have used an inferior function (less developed and unconscious) as a coping or supporting resource to externalize the suffering during the crisis triggered by cancer.

This research, being a small part of the patient’s diagnosis, is limited to only describing the findings found in patients with esophageal cancer. The possibility of intervening with the patient to integrate unconscious aspects, while not disregarding the reality of the external world so frequent in introverted people, would be a suggestion for future work on psychological typology, through for example, directed imagination using relaxation techniques. In addition, we only studied one group of patients, meaning that there is the possibility of other findings in patients with other types of neoplasia, as well as in another illness group.

As part of the multidisciplinary team that treats cancer patients, we believed that the characterization of these patients’ personalities was of great value, because it was the beginning of a way to care for the patient based on their interests and way of life and not through the specific management of the hospital professionals.

## CONCLUSIONS

Introversion was the prevailing attitude of the participants of this research. The most outstanding psychological type was an introverted sensation, with feeling as a secondary function (43.33%), and second most being the introverted feeling type, with sensation as a secondary function (24.44%).

Therefore, when attending these patients, health professionals must have a practical and objective posture, seeking to guide the patients according to their reality. In addition, they should be empathetic in their manner, establishing a significant bond while respecting their limits, especially when it comes to their reserved and introspective ways of interacting with the world and the people around them.
